# Clinical Validation of Fetal cfDNA Analysis Using Rolling-Circle-Replication and Imaging Technology in Osaka (CRITO Study)

**DOI:** 10.3390/diagnostics11101837

**Published:** 2021-10-04

**Authors:** Ritsuko Kimata Pooh, Chika Masuda, Risa Matsushika, Megumi Machida, Takako Nakamura, Masayoshi Takeda, Hiroyasu Ohashi, Mami Kumagai, Kohtaro Uenishi, Fredrik Roos, Fredrik Persson, Osamu Shimokawa

**Affiliations:** 1 Clinical Laboratory, Ritz Medical Co., Ltd., Osaka 543-0001, Japan; masuda.chika@ritz-med.com (C.M.); matsushika.risa@ritz-med.com (R.M.); takeda.masayoshi@ritz-med.com (M.T.); ohashi.hiroyasu@ritz-med.com (H.O.); kumagai.mami@ritz-med.com (M.K.); uenishi.koutarou@fetal-medicine-pooh.com (K.U.); shimokawa.osamu@ritz-med.com (O.S.); 2 CRIFM Prenatal Medical Clinic, Osaka 543-0001, Japan; machida.megumi@fetal-medicine-pooh.com (M.M.); nakamura.takako@fetal-medicine-pooh.jp (T.N.); 3 Vanadis Diagnostics, PerkinElmer Inc., 191 38 Sollentuna, Sweden; Fredrik.Roos@PERKINELMER.COM (F.R.); Fredrik.Persson@PERKINELMER.COM (F.P.)

**Keywords:** noninvasive prenatal genetic testing, NIPT, placenta, fetus, ultrasound, nuchal translucency, cell-free DNA, rolling-circle-replication, imaging technology

## Abstract

Background: Noninvasive prenatal genetic testing (NIPT) has been adopted as the first choice for aneuploidy screening. The purposes of this study were to investigate the accuracy of Vanadis^®^ NIPT (hereafter CRITO-NIPT) in order to gain a deeper insight into the reasons for discrepancies, as well as to discuss the role of fetal ultrasound. Methods: Between 2019 and 2020, CRITO-NIPT was performed in 1218 cases of patients who underwent CVS or amniocentesis after a detailed fetal ultrasound exam and genetic counseling. The CRITO-NIPT results were compared with the genetic results. In cases of test discrepancies, the placentae were collected for detailed genetic research, and the pre-procedure fetal ultrasound findings were referred to. Results: The positive predictive value of T21, T18, and T13 was 93.55%, 88.46%, and 100%, respectively. In 90% of the of false positive (FP) cases, the placentae were examined. In 75% of the CRITO FP-T21 cases, placental mosaicism, or a demised twin’s T21, were confirmed. There were complicated mosaic cases, including tetrasomy 21/trisomy7 and monosomy 21/trisomy21 cases. In one of three no-call cases, an intermediate deletion of chromosome 13 was detected. Conclusions: The CRITO study investigated the mechanism of false positives, and the detailed mechanisms in mosaic and no-call cases. There have hitherto been no reports that have provided insight by partitioning the placenta to compare the NIPT and invasive test results, nor that have provided detailed ultrasound findings in the cases of discordant results, revealing the demonstrated importance of, and necessity for, detailed ultrasonography. This article describes the potential of rolling-circle replication as a powerful biosensing platform, as well as the importance of examining the fetus in detail with ultrasound. However, we should remember that the potential applications raise ethical and social concerns that go beyond aneuploidy and its methodology.

## 1. Introduction

Prenatal aneuploidy screening using fetal cell-free DNA (cfDNA) in maternal plasma, also known as noninvasive prenatal genetic testing (NIPT), is rapidly being adopted as the first choice for aneuploidy screening. The primary method of the NIPT currently used is next-generation sequencing (NGS), and there have been reports on expanded NIPT, the detection of copy number variations (CNV) [1,2], and single-gene mutations of rare diseases [3,4], for fetal cfDNA in maternal plasma. However, the positive predictive value of fetal cfDNA testing in maternal plasma for microdeletion and microduplication syndromes is lower than that for autosomal trisomy [5,6], and the question of whether screening with the expanded cfDNA approach is necessary must be carefully debated [7,8]. Considering the practicality of NIPT in general obstetric practice, the possibility that NIPT might detect maternal CNV [9] or aneuploidy due to maternal malignancy [10], as well as whether the detection by NIPT of diseases that are difficult to confirm by invasive testing is really consistent with clinical needs, careful consideration should be given to the different dimensions of general clinical practice and advanced medicine and research. Recalling that the original purpose of NIPT in clinical practice was to screen for major congenital diseases, it is necessary to use an efficient and simple method that narrows down the test targets and reduces the TAT (turnaround time), cost, and labor.

In all NIPT tests, there will be false positives, false negatives, and no-call cases [11,12] due to natural biological issues such as vanishing twins, the existence of confined placental mosaicism (CPM), and somatic mutation mosaicism.

The Vanadis^®^ NIPT System [13,14] (Vanadis Diagnostics, PerkinElmer, Sollentuna, Sweden), which is used in this study, consists of three steps: the extraction, the core, and the view. Unlike conventional NIPT systems, the Vanadis^®^ NIPT System eliminates PCR bias and the need for sequencing, elaborate sample preparation and complex bioinformatic analyses. This unique system uses two powerful innovations, rolling-circle replication and digital imaging technology, to detect fetal aneuploidy using cfDNA in maternal plasma, targeting fetal chromosomes 13, 18, and 21. The procedure is simple and mostly automated. As a result, Vanadis^®^ has successfully reduced costs and the need for additional human resources.

Our study has been named the CRITO study, an acronym for the clinical validation of fetal cfDNA analysis using rolling-circle-replication and imaging technology in Osaka. The CRITO study is an observational prospective clinical validation study that was conducted in Osaka, Japan, using the Vanadis^®^ NIPT assay. This study provides, not only statistical data of the Vanadis^®^ NIPT assay (hereafter referred to as CRITO-NIPT), but also detailed genetic testing by chorionic villus sampling (CVS) and amniocentesis (AC) in all cases. Furthermore, the placentas of the cases with inconsistent CRITO-NIPT and genetic test results were collected from the birthing centers, and detailed placental genetic results were obtained. This information along with the ultrasound findings performed prior to genetic testing, allowed us to provide a comprehensive and detailed analysis.

The objectives of this study were: to validate the accuracy of the Vanadis^®^ NIPT system; to determine the causative factors and mechanisms of discrepancies between CRITO-NIPT and genetic test results by examining the parental blood, placenta, umbilical cord, and membranes; and to examine the usefulness and significance of fetal ultrasound before genetic testing by examining the fetal ultrasound findings in these discrepant cases.

## 2. Materials and Methods

Between April 2019 and March 2020, 1218 pregnant women who underwent CVS or AC at the Fetal Diagnostic Center of the CRIFM Prenatal Medical Clinic were enrolled in this study. After a detailed fetal ultrasound scan and genetic counseling, CVS was performed in 1077 women (88.42%), and AC was performed in 141 women (11.58%). After obtaining informed consent, approved by the Institutional Review Board (CRI-IRB-012, with final approval on 23 March 2019), maternal blood was drawn in two cell-free DNA blood collection tubes (Streck, Omaha, NE, USA) immediately before an invasive procedure. Blood samples were transferred within two hours to the laboratory, where the CRITO-NIPT assay was performed. The inclusion criteria were pregnant women at least 18 years old, with a gestation period of at least 11 weeks. The exclusion criterion was a higher-order pregnancy.

The mean maternal age of all 1218 patients was 36.2 (18–50), and the mean body mass index (BMI) was 21.3 (13.0–36.8). The chorion, amnion, and fetus were detected by fetal ultrasound. In 1218 cases, 95.7% were singletons, 1.81% were dichorionic diamniotic (DCDA) twins, 0.82% were monochorionic diamniotic (MCDA) twins, and other cases were associated with vanishing twins, as shown in Table 1.

Prior to genetic testing, all participants underwent detailed fetal ultrasonography examinations. The equipment used in this study was a VOLUSON E10 with a transabdominal 3D transducer, and 6–12 MHz transvaginal 3D transducers (GE Healthcare, Milwaukee, WI, USA). The chorionic villi and amniotic cells were examined by various methods, including quantitative fluorescent polymerase chain reaction (QF-PCR); G-band in all cases; fluorescence in situ hybridization (FISH) in cases with a suspicion of chromosomal mosaicism; single nucleotide polymorphism (SNP) microarray in selected cases with a suspicion of fetal abnormality; and targeted exome sequencing in selected cases with a suspicion of genetic diseases. The testing kits used in this study were: the Aneufast™ QF-PCR Kit (Genomed Ltd., Kent, UK) for QF-PCR; the AneuVysion Multicolor DNA probe kit (Vysis, Downers Grove, IL, USA) for FISH; CytoScan^®^ HD Arrays (Affymetrix, Santa Clara, CA, USA) for the SNP microarray; and the TruSight One Sequencing Panel (Illumina, San Diego, CA, USA) for target exome sequencing. The Vanadis^®^ system [13,14] used in the CRITO study has two features: rolling-circle-replication and imaging technology. The detailed technology has been described previously [13]. Fetal cfDNA extracted from maternal plasma is subjected to specific fragmentation by restriction enzymes. The cfDNA fragments are hybridized to probes designed to form circular DNA complexes. The DNA circles are copied approximately 1000 times by rolling-circle-replication to generate rolling-circle-replication products (RCPs). Each RCP contains the approximately 1000 copies of a chromosome specific tag that is recognized by corresponding fluorescently labeled oligonucleotides. These labeled RCPs are deposited on a nanofilter microplate, and the deposited RCPs are finally imaged using the Vanadis View imaging system [13].

**Figure 1:**
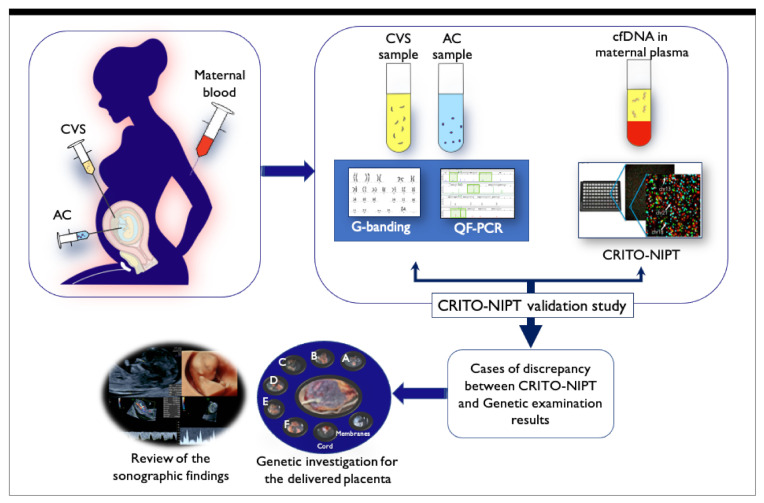
Schematic illustration of the CRITO study. Maternal blood was drawn for CRITO-NIPT immediately before CVS or amniocentesis, and a validation study was conducted to compare the CRITO results with the CVS/AC genetic results. Next, in CRITO false-positive, CRITO false-negative, CRITO no-call, and CVS/AC mosaic cases, the placentas at delivery were collected and divided for genetic research, and the fetal ultrasound results performed before the invasive test were referred to.

The CRITO-NIPT diagnostic accuracy was measured. The sensitivity, specificity, positive predictive value (PPV), and negative predictive value (NPV) were measured for T21, T18, and T13. In cases of a false positive (FP), false negative (FN), or no-call by CRITO-NIPT, and mosaicism cases by genetic exams, the genetic results of placentae and parental blood, as well as prior ultrasound (US) findings, were investigated, as shown in Figure 1. The whole placentas collected from the delivery hospitals were fractionated into four or six sections. All placental parts, the membranes, and the umbilical cord were examined by the QF-PCR, FISH, and G-band methods and, in specific cases, the mechanism of discordancy was speculated. The sonographic findings prior to genetic testing were compared to all test results in order to examine the usefulness of the ultrasound examination.

## 3. Results

### 3.1. Genetic Profile of 1218 Cases

The genetic exams of CVS and AC resulted in 975 normal cases (80.05%), 59 normal variant cases (4.84%), and 184 abnormal genetic cases (15.11%). The breakdown of these 184 cases is shown in Table 2. Trisomy 21 was confirmed in sixty cases (32.6%); Trisomy 18 in forty-seven cases (25.5%); Trisomy 13 in twelve cases (6.5%); chromosome 21-, 18-, and 13-relevant mosaicism in seven cases (3.8%); sex chromosome aneuploidies (SCA) in sixteen cases (8.7%); triploid in one case (0.5%); non-21, 18, and 13 aneuploidy mosaicism in twelve cases (6.5%); structural abnormality in five cases (2.7%); and structural abnormality mosaicism in one case (0.5%). Chromosomal microarray (CMA) was performed in eighty-five cases, and pathogenic results were detected in ten cases (5.4%). Targeted exome sequencing (for 4813 genes) was performed in thirty-seven cases, and single-gene mutations were detected in thirteen cases (7.1%), as shown in Table 2.

### 3.2. Measures of CRITO-NIPT Diagnostic Accuracy

In a total of 1208 cases, after excluding seven cases of trisomy-mosaicism detected in CVS samples, and three no-call cases, the sensitivity of T21, T18, and T13 was 98.31%, 100%, and 100%, respectively; the specificity was 99.65%, 99.48%, and 100%, respectively; the PPV was 93.55%, 88.46%, and 100%, respectively; and the NPV was 99.91%, 100%, and 100%, respectively (Table 3).

Among twenty-two cases of DCDA twins, there were four cases with genetic discordancy, two cases with T21/normal, and the other two cases with T18/normal. In all four cases, CRITO-NIPT resulted in positive trisomies. There were no trisomy cases in ten MCDA twins and three MCDA vanishing twins with an empty second sac. Out of twelve DCDA vanishing twins with an empty second sac, one had a genetic discordancy of T18/normal, and CRITO-NIPT resulted in T18. In five cases of DCDA vanishing twins with the second sac containing a nonviable fetus, all viable fetuses had normal karyotypes, but CRITO-NIPT resulted in positive T21 in two cases.

### 3.3. Placental Investigation Results in Cases with Discordancy between CRITO-NIPT and Genetic Results

**Figure 2:**
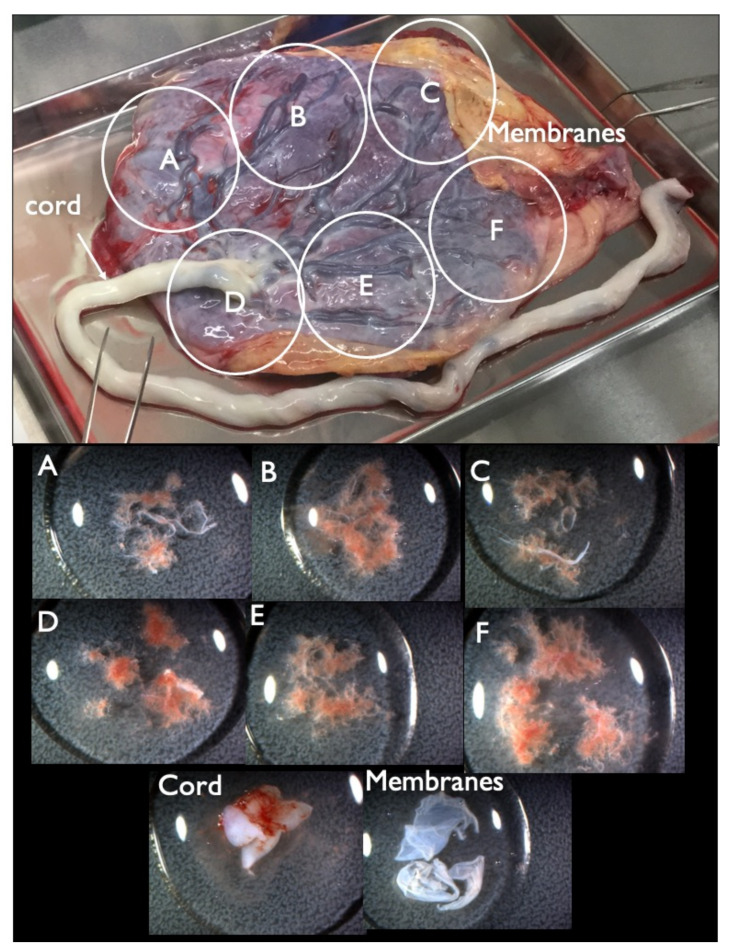
Collected placenta and tissues from each part. The placenta was divided into four or six parts (upper figure, A–F parts) and tissue from each A–F parts (lower figure) was extracted from each A–F part, along with the cord and membrane tissue for further genetic tests.

FP-T21 was found in four cases, FN-T21 was found in one case, and FP-T18 was found in six cases. There were no cases of FN-T18, FP-T13, or FN-T13. Nine placentas were collected at delivery, covering all four FP-T21 cases, and five out of six FP-T18 cases. Each placenta was divided into four or six parts, and tissue was extracted from each part, along with the cord and the membrane tissue, as shown in Figure 2, and all tissues were genetically tested. The FP and FN cases are listed in Table 4.

#### 3.3.1. False Positive (FP) CRITO-NIPT

**Figure 3:**
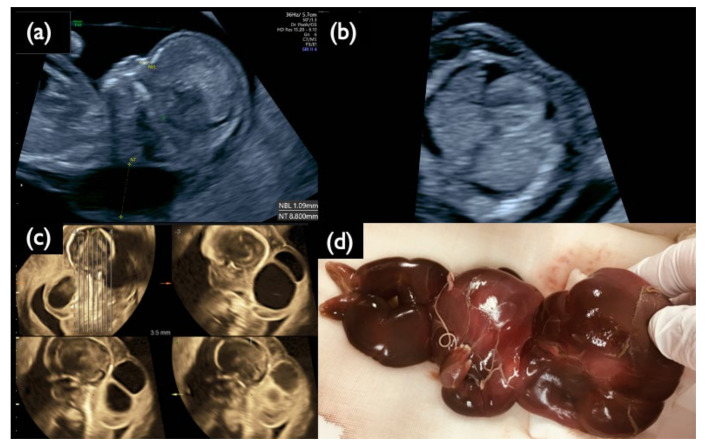
Sonographic findings at 11^+6^ weeks, and 17 weeks, and IUFD fetus after delivery in Case FP1. (**a**) Mid-sagittal section of the fetal head. Nuchal translucency (NT) of 8.8 mm and a small nasal bone are demonstrated. (**b**) Horizontal section of the thoracic area. Pleural effusion is visualized bilaterally. (**c**) 3D tomographic ultrasound image in the sagittal section with a coronal guide section (left upper). General edema and large cystic hygromas are shown. (**d**) Picture of the dead fetus on delivery at 20 weeks. The fetus died in utero at 19 weeks of gestation.

**Figure 4:**
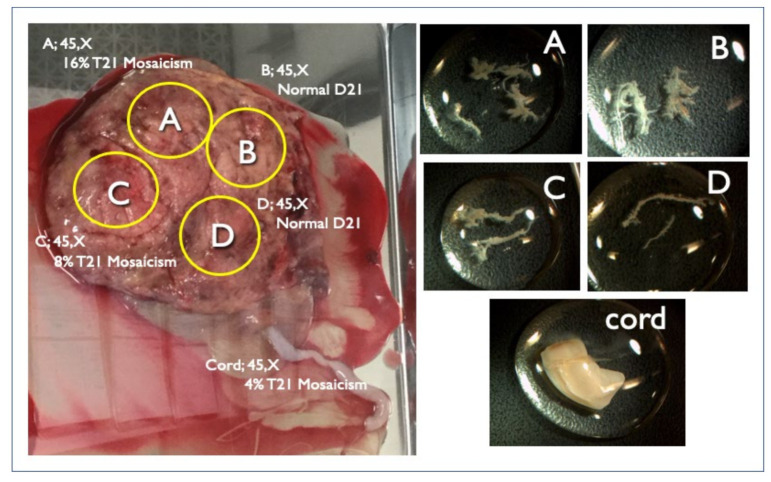
Genetic results of the placenta and cord delivered at 20 weeks in Case FP1. Intrauterine fetal demise occurred at 19 weeks due to hydropic Turner syndrome. The left figure is the placenta with the FISH results. Right figures are each specimen of A, B, C, and D chorionic villi and cord from each placental A, B, C, D part and cord (left figure) respectively for genetic investigation.

**Figure 5:**
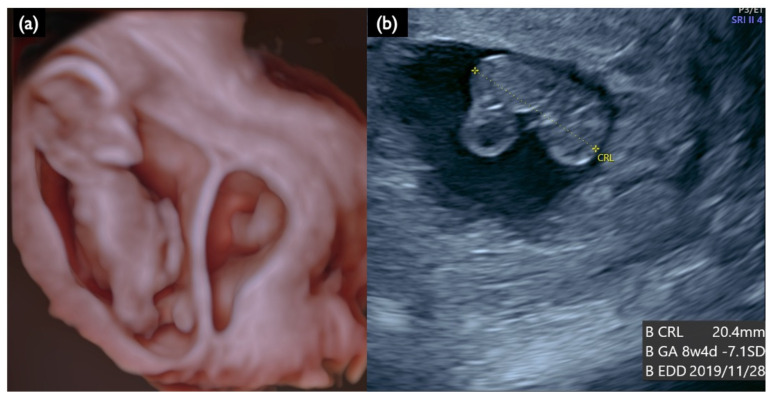
Sonographic findings at 13 weeks and 4 days of a DCDA vanishing twin with the second sac containing a nonviable fetus (Case FP2). (**a**) 3D reconstructed image. Small dead embryo exists in the smaller gestational sac. (**b**) Crown lump length (CRL) of a nonviable fetus was 20.4 mm, compatible with 8 weeks and 4 days.

**Figure 6:**
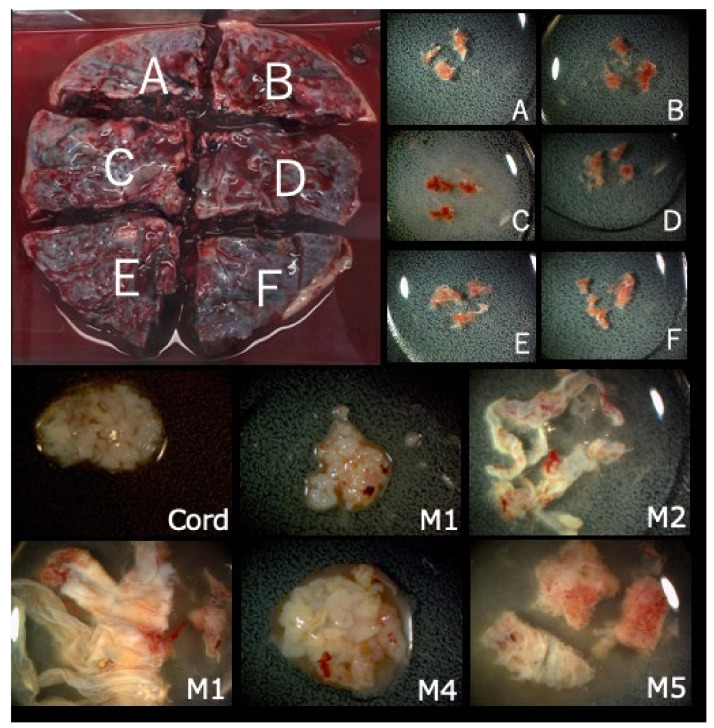
Segmentation of placenta and membranes delivered at term in Case FP3.

There were four FP-T21 and six FP-T18 cases. We collected nine placentas (90%) from nine different hospitals. One FP-T18 placenta could not be collected. All tissues from the divided placental parts, the umbilical cord, and the membranes were examined by the QF-PCR, FISH, and G-banding methods. Maternal blood was also tested in order to investigate the presence or absence of maternal trisomy mosaicism. In three out of four FP-T21 cases (75%), T21 mosaicism was found from the placenta or membranes. In the remaining case ultrasound findings point to the nonviable fetus may be T21. In Case FP1, a fetal ultrasound at 11 weeks of gestation showed an increased NT (8.8 mm), pleural effusion, and tachycardia, and was strongly suggestive of Turner syndrome (Figure 3). The sex chromosome abnormality of 45,X was detected in the CVS sample and all placenta sites, but a different frequency of the T21 mosaicism was detected in about half of the placenta, and the cord, and so confined placental mosaicism (CPM) was determined as the cause of the FP-T21 (Figure 4). In Case FP2, the placental investigation did not show anything abnormal. However, based on the ultrasound findings, as shown in Figure 5, it was inferred that the cause of FP-T21 may be due to the nonviable twin being T21. In Cases FP3, a DCDA vanishing twin, with the second sac containing a nonviable fetus, was detected by fetal ultrasound. The placental investigation resulted in the confined membranous mosaicism of T21 (Table 5 and Figure 6), indicating that a nonviable T21 twin caused the FP-T21. In Case FP4, a T21 CPM was found in a sixth of the placenta, which was determined to be the cause of the FP-T21.

In all five FP-T18s, T18 was not found in any part of the placenta, membranes, or cord (Table 4). In ten cases with FPs, a prior ultrasound did not detect an increased NT in nine cases (90%), and only one case with an increased NT (FP1) was proven as Turner syndrome.

#### 3.3.2. False Negative CRITO-NIPT

**Figure 7:**
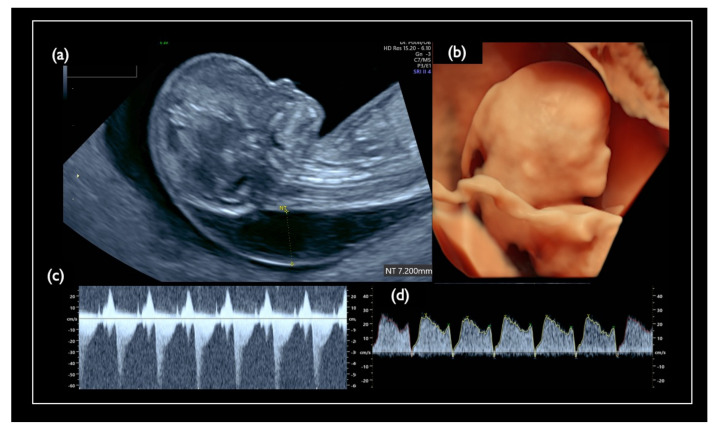
Pre-procedure sonographic findings in Case FN1. (**a**) Mid-sagittal section of the fetal head. Nuchal translucency (NT) was 7.2 mm, and the nasal bone is not visualized. (**b**) 3D reconstructed fetal face. Flat profile with the low-set ear is detected. (**c**) Tricuspid regurgitation. (**d**) Tiny reversed end-diastolic flow of the ductus venosus.

In our case series, we had only one case with FN-T21 (Case FN1 in Table 4). CRITO-NIPT was negative with a Z-score of 1.07, but the CVS resulted in T21 in all cells. The prior US findings indicate a strong suspicion of T21, as shown in Figure 7, including an increased NT of 7.2 mm, a nasal bone defect, a low-set ear, swollen eye bags, reversed blood flows at the tricuspid valve, and ductus venosus. The pregnancy was terminated at 13 weeks, and we could not obtain the placenta of this case.

### 3.4. Investigation of Chromosome 21-, 18-, and 13-Relevant Mosaicism

We had seven cases with chromosome 21, 18, and 13-relevant mosaicism (Table 6). In seven mosaicisms, there were two complicated mosaic cases (Case Mo2 and Mo4).

**Figure 8:**
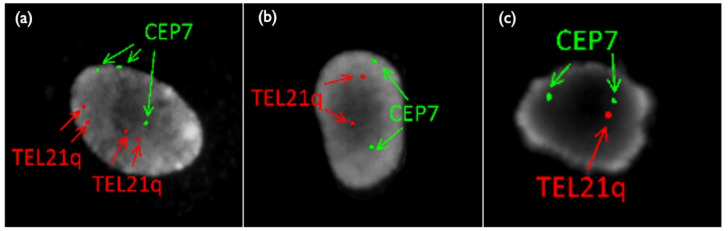
FISH results of placental sample in Case Mo2. (**a**) Tetrasomy 21/Trisomy7 (**b**) Disomy21/Disomy 7 (**c**) Monosomy21/Disomy 7. TEL21q: telomere probe for 21 long-arm, CEP 7: centromere (D7Z1) probe for chromosome 7.

Case Mo2 was a 50-year-old mother, with normal NT thickness by ultrasound, and negative CRITO-NIPT with a Z-score of 1.11. CVS resulted in tetrasomy21 + trisomy7 mosaicism, and the karyotype was 49,XX,+7,+21,+21/46,XX (72%/28%). The parents did not undergo further examination by amniocentesis and delivered a healthy 2928 g female baby at term. As is shown in Table 7 and Figure 8, the placental investigation revealed the mosaicism of tetrasomy21 + trisomy7, monosomy21 + disomy7, and normal cells. The cord and cord blood showed normal disomy in all cells, and confined placental mosaicism was confirmed.

**Figure 9:**
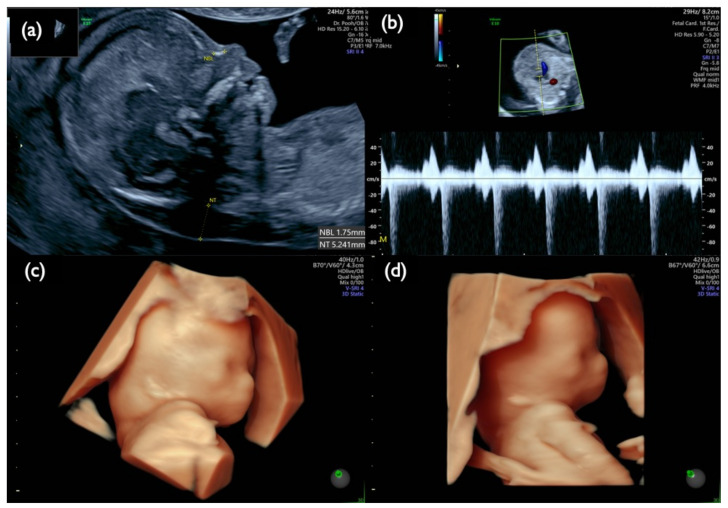
Fetal ultrasound scan of Case Mo4 at 13 weeks. (**a**) Mid-sagittal section of the fetal head. Nuchal translucency (NT) of 5.2 mm and small nasal bone are demonstrated. (**b**) Tricuspid regurgitation. (**c**,**d**) 3D reconstructed fetal face. A flat profile with a low-set ear and swollen eye bags are clearly demonstrated.

Case Mo4 had inconsistent results regarding CVS, AC, the placenta, and the cord blood. A 23-year-old mother underwent a first-trimester ultrasound scan that showed a high risk of T21, with an NT of 5.4 mm, tricuspid regurgitation (TR), a low-set ear, and swollen eye bags, as shown in Figure 9. The CRITO-NIPT result was T21-positive with a Z-score of 10.87. CVS-QF-PCR suspected T21 mosaicism, FISH showed a complex mosaicism of Monosomy(M) 21 (72%), Disomy(D) 21 (20%), and T21 (8%), and the G-band resulted in 45,XX,−21 in all 50 cells. The subsequent AC-QF-PCR result was T21, FISH showed mosaicism with M21 (1%), D21 (8%), and T21 (91%), and G-band resulted in T21 with Robertsonian translocation (46,XX,+21,der(21;21)(q10;q10)) in all 20 cells. The parental blood exam showed normal karyotypes of both the mother and the father. The pregnancy was terminated at 20 weeks. We collected the placenta, divided it into four parts, and examined all the placental parts, the membranes, and the umbilical cord by various genetic methods, as shown in Table 8. While the placental karyotype was 95.5% of M21, and 4.5% of Robertsonian T21, the membranes showed 2% of M21 and 98% of Robertsonian T21, and the cord showed 100% of Robertsonian T21.

**Figure 10:**
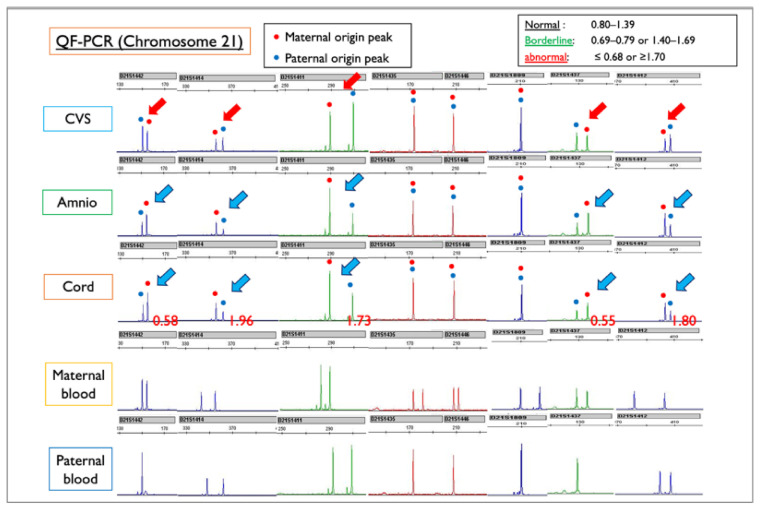
QF-PCR results for each sample of Case Mo4, compared with the maternal and paternal blood sample. CVS: chorionic villus sample; Amnio: amniotic fluid sample. Red figures ≤0.68 or ≥1.70 indicate Trisomy 21. In this case, maternal and paternal peak patterns show the normal disomy of chromosome 21. In the amniotic fluid and cord samples, the blue arrows indicate that the peak of chromosome 21 of maternal origin is higher than that of paternal origin, a finding that is suggestive of T21. In contrast, in the CVS sample, the red arrows indicate that the peak of the mother-derived chromosome 21 is lower than that of the father-derived chromosome 21, suggesting the presence of more M21 than T21. From the QF-PCR analysis, we concluded that the Robertsonian T21 was created as an isochromosome with a duplicated long arm of maternal chromosome 21.

We used QF-PCR to determine whether the Robertson translocation originated from the father or the mother. Trio QF-PCR analysis was conducted after obtaining maternal and paternal blood samples (Figure 10). In the amniotic fluid and cord samples, the peak of chromosome 21 of maternal origin was higher than that of paternal origin, and this finding indicated a strong suspicion of T21. In contrast, in the CVS sample, the peak of the maternal chromosome 21 was lower than that of the paternal ch21, suggesting the presence of more M21 than T21. As a result of QF-PCR analysis, we concluded that the Robertsonian translocation formed an isochromosome with duplication of a long arm in one of the maternal chromosomes 21. In other words, one of the mother’s chromosomes 21 was doubled, and the translocation was not between homologous chromosomes 21, but isochromosome 21, and the karyotype was rewritten as 46,XX,i(21)(q10)(q10) instead of 46,XX,+21,der(21;21)(q10;q10).

**Figure 11:**
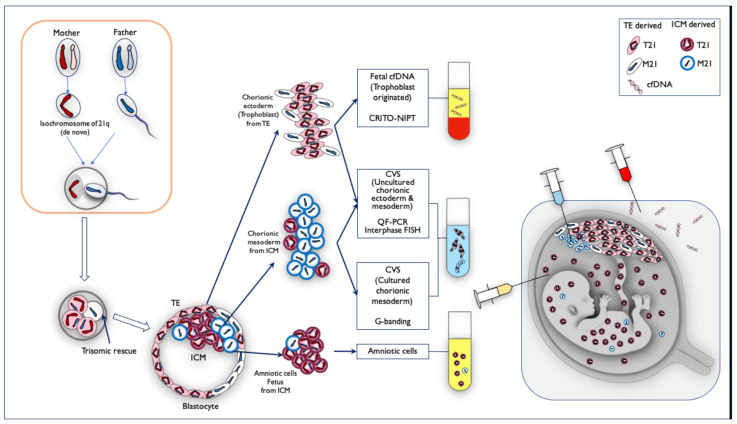
The mechanism of mosaicism in Case Mo4, in which the frequency of monosomy/disomy/trisomy mosaicism differed among specimens. First, the oocytes of de novo isochromosome 21 were formed during the meiosis of the mother, and the fertilized eggs became T21 with the mother-derived isochromosome 21. The M21 cell line arose because of trisomic rescue at the early stage before the blastocyst. Both trophectoderm (TE) and the internal cell mass (ICM) in the blastocyst became mosaicism of Robertsonian T21 and M21. On the basis of the genetic results, we estimated the mosaic ratio. TE-derived chorionic ectoderm (trophoblast) was probably Robertsonian T21-dominant, and fetal cfDNA is derived from trophoblast, so CRITO-NIPT was T21-positive. The DNA from uncultured cells in the CVS samples was a mixture of both uncultured chorionic ectoderm from TE (T21 predominant), and chorionic mesoderm from ICM (M21 predominant). The cultured chorionic mesoderm was a mosaicism of predominantly M21-cells in ICM. Therefore, the CVS G-banding result was M21, which was completely different from the CRITO-NIPT result of T21. On the other hand, amniotic fluid cells and fetal cells were derived from T21-dominant ICM sites, which may have led to the Robertsonian T21 results.

From all of the genetic results, and the fact that the Robertsonian translocation of the isochromosome originated from the mother, we estimated the mechanism of the discordant mosaicism ratio among the fetal cfDNA, QF-PCR, and the interphase FISH of CVS, cultured CVS, and amniotic samples, illustrated in Figure 11. First, the oocytes of de novo isochromosome 21 were formed during the meiosis of the mother, and the fertilized eggs became T21 with the mother-derived isochromosome 21. The M21 cell line arose because of trisomic rescue at the early stage before the blastocyst. Both the trophectoderm (TE), and the internal cell mass (ICM), in the blastocyst became a mosaicism of Robertsonian T21 and M21. Eventually, M21s were present in some part of the trophoblast and most of the chorionic mesoderm. Most of the ICM containing Robertsonian T21 cells became the amniotic cells and fetus.

**Figure 12:**
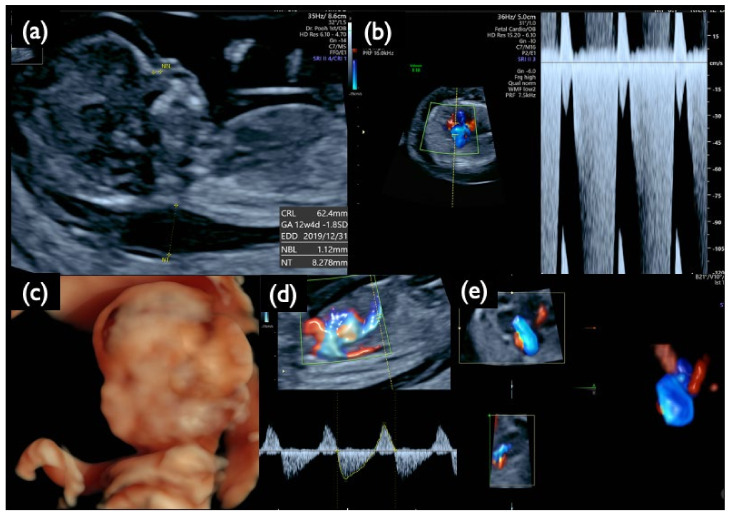
Sonographic findings at 13 weeks and 5 days of gestation in Case Mo5 with T18 mosaicism and a negative CRITO-NIPT result. (**a**) Mid-sagittal section of the fetal head. Nuchal translucency (NT) of 8.3 mm, a small nasal bone, and micrognathia are demonstrated. (**b**) Severe tricuspid regurgitation blood flow. (**c**) 3D reconstructed image of fetal face and upper extremities. Micrognathia, a low-set ear, and wrist contracture are visualized. (**d**) Reversed end-diastolic flow of ductus venosus. (**e**) 4D cardiosonographic image. Cardiomegaly with outflow tract abnormality is demonstrated. Intrauterine fetal demise was confirmed thereafter.

In Case Mo5, a fetal ultrasound prior to genetic testing demonstrated an increased NT of 8.3 mm, a small nasal bone, micrognathia, a low-set ear, reversed flow waveforms of the tricuspid valve and ductus venosus, cardiomegaly with congenital heart disease, a single umbilical artery, and other findings listed in Table 6 and shown in Figure 12. These images suggest the characteristic findings consistent with T18: CRITO-NIPT was negative, but the FISH results of the uncultured cells from CVS revealed 56% mosaic T18, and G-band showed 44% mosaic T18.

### 3.5. No-Call (NC) CRITO-NIPT

**Figure 13:**
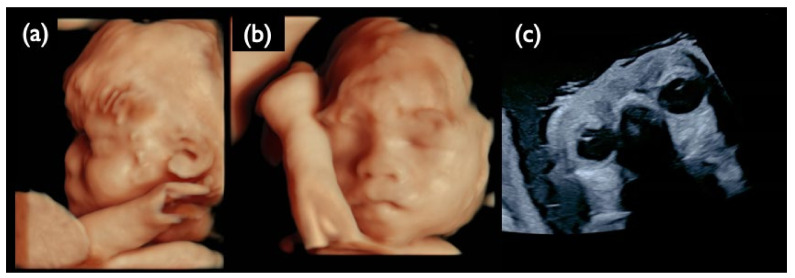
Ultrasound images at 30 weeks of gestation in the NC1 case. (**a**) 3D ultrasound image of the fetal profile. (**b**) 3D ultrasound image of the frontal face of the fetus. (**c**) Ultrasound image of fetal eye sand lenses on both sides. All images show that the fetus is hypertrichotic and has no ocular lesions at this gestational age.

**Figure 14:**
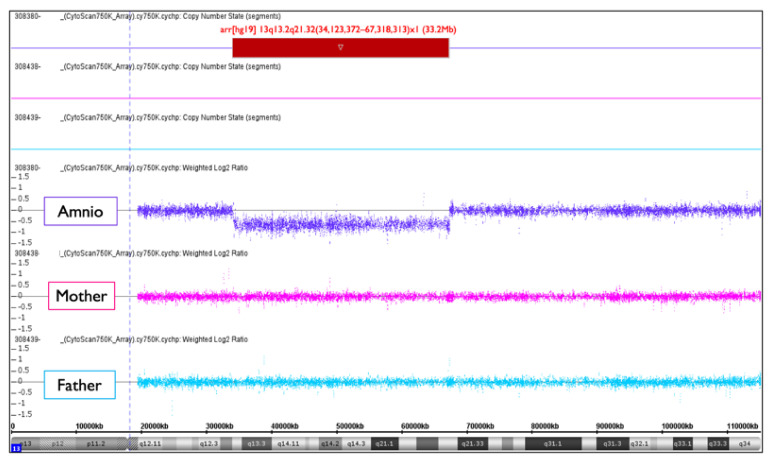
Trio SNP microarray result of NC1. Amniotic cells (amnio) show arr[hg19] 13q13.2q21.32(34,123,372−67,318,313)x1 (33.2Mb). It must be de novo intermediate deletion because both the mother and the father had a normal chromosome 13.

No-call CRITO-NIPT results were found in three cases, with T21, T18, and the intermediate partial deletion of 13q (Table 9). All of the mothers had normal BMIs of 21.1, 22.8, and 25.8, and no maternal complications. In Case NC2 with T21, and Case NC3 with T18, the fetal ultrasound demonstrated increased NT and the characteristic features of each trisomy, as listed in Table 9. We did not find the causal factors for no-call NIPT in these two cases. Case NC1, a 28-year-old mother, was first referred because of sonographic findings at 30 weeks, including hypertrichosis, a strongly suspected genetic disorder, as shown in Figure 13. The AC resulted in a 13q13.2-q21.32 intermediate partial deletion. The origin and the exact cutting points were examined by an SNP microarray (Figure 14), and de novo intermediate deletion, arr[hg19] 13q13.2q21.32(34,123,372–67,318,313)x1, sized 33.2 Mb, was confirmed.

The deleted section of 13q13.2–q21.32 includes the *RBI* gene, which causes retinoblastoma. The sonography at 30 weeks showed normal eyeballs and lenses (Figure 13c), but retinoblastoma developed two months after birth, and an ophthalmectomy was performed. It may be recommended to proceed with invasive testing using chromosomal microarrays in no-call cases, taking into account the possibility of partial deletions.

## 4. Discussion

### 4.1. Measures of CRITO-NIPT Diagnostic Accuracy

This CRITO study investigated, not only the mechanism of false positives, but also the detailed mechanisms in mosaic and no-call cases. There have hitherto been no reports that provide insight by dividing the placenta in order to compare NIPT and invasive test results, nor that provide detailed ultrasound findings in cases of discordant results. Currently, NIPT is expanding to microdeletion syndromes and single gene disorders, but even for the three basic trisomies, there can be complex mechanisms, as shown in this paper. In addition, the importance and necessity of detailed ultrasonography was demonstrated.

In this study, the mean maternal age of all 1218 patients was 36.2 (18–50), and all patients underwent invasive tests because of an advanced maternal age, abnormal ultrasound findings, or other indications. In other words, the CRITO study was conducted for a high-risk population. However, it has been reported that there is no significant difference in the percentage of fetal cfDNA according to the risk classification of the patient in clinical practice [15]. Our CRITO study showed a high sensitivity and specificity in all three trisomies. Additionally, the PPV of our study was higher in all trisomies than that reported using the NGS method in the same region [16].

### 4.2. False Positive and False Negative CRITO-NIPT

The reasons for discordant NIPT results may be due to maternal CNV [9], mosaicism, fetal mosaicism, maternal cancer [10,17], CPM, or a vanishing twin [18]. Bianchi et al. [10] first reported false-positive NIPT resulting from the presence of maternal malignancy. Because all cancers have somatic gene mutations that may be reflected in circulating cfDNA, and because these aneuploidies include the aneuploidies of chromosomes 21, 18, and 13, which are the targets of NIPT, discrepant NIPT results, such as false positives or no-calls, may be explained by the presence of maternal malignancy [17]. In this study, no maternal tumors were found according to maternal follow-ups, but maternal malignancy should be considered in false-positive and no-call cases. In our case series, in three out of the four FP21 cases, the causes of the FPs were determined by placental examination, and in the remaining case, it was suggested that the nonviable twin may have been T21.

### 4.3. Chromosome 21-, 18, and 13-Relevant Mosaicism

Many of the NIPT-related mosaics are false-positive cases because of placental mosaicism [18,19,20]. In our case series, Mo4 showed a discrepancy between the CRITO results of T21 and the CVS results of M21, indicating that Robertsonian T21 was involved in this very rare case. In a report by Nguyen et al. [21], a mosaic of isochromosome-type Trisomy 21, and trisomy-rescued Monosomy 21, was detected in the infant, which is the same cell line mosaic as in Case Mo4. We obtained completely discordant results by different genetic tests. CRITO-NIPT that reflected only the trophoblast of uncultured CVS was T21-positive, QF-PCR and uncultured FISH that reflected both the trophoblast of uncultured CVS, and the chorionic mesoderm of cultured CVS, showed a mosaic T21, CVS-karyotyping that reflected the chorionic mesoderm of cultured CVS showed M21, and amniotic cell-karyotyping showed T21. We also added genetic analyses using the placenta after delivery, and all the genetic tests of the different samples revealed that Robertsonian T21 was a de novo isochromosome of maternal origin, and the subsequent production of monosomy was due to trisomic rescue, as shown in Figure 8. We presumed that trisomy rescue occurred before the blastocyst stage, and monosomy cells were generated. If trisomic rescue occurs at an earlier stage, a greater proportion of monosomy 21 cells may lead to an early embryonal death.

In our results, shown in Table 8, the FISH result shows a mosaicism of M21/D21/T21. The origin of the two signals of LSI21 in uncultured FISH is considered to be the result of the fact that the signals of Robertson type 21 in Trisomy 21 cells were closely adjacent to each other, and three signals seemed to be two signals.

### 4.4. No-Call NIPT

Although historically, the no-call rates of NIPT were approximately 3–5% [14], with the Vanadis system, we only had a no-call rate of 0.2%. It has been reported that a no-call NIPT is often caused by an insufficient percentage of fetal cfDNA in cases of heavy maternal weight [22], preeclampsia [2], or gestational diabetes [23]. However, Kruckow et al. [24] showed no significant relations between maternal BMI and the fetal cell number in maternal circulation.

In one case (Case NC1) of no-call CRITO-NIPT, it was assumed that the Vanadis^®^ NIPT system could not discriminate aneuploidy because of the intermediate deletion of chromosome 13. The system simultaneously checks whether the number of RCPs obtained from each chromosome is reliable when calculating the Z-score. Specifically, the system refers to the relative amount of all investigated autosomes by looking at the 21/13, 21/18, and 13/18 ratios, to assess whether the result is consistent with a disomy or trisomy.. If partial monosomy is present among chromosomes 13, 18, and 21, the number of RCPs for that chromosome will be reduced, and thus the balance between the chromosomes altered in a way not consistent with a disomy or trisomy. In the case of NC1 in this study, due to the intermediate deletion of chromosome 13, the Vanadis^®^ NIPT system judged the data to be beyond the expected acceptable range, and the final result was a no-call.

### 4.5. Significance of Fetal Sonography Prior to Genetic Testing

In twenty-one cases with discrepant results between the CRITO-NIPT and genetic results, fetal sonographic findings, including NT measurements, were mostly consistent with final diagnoses. In nine (90%) of the ten FP cases, normal NT was found, and the remaining case (Case FP1) showed increased NT, and the highly suspected Turner syndrome sample was indeed proven to have a 45,X karyotype. In Case FN1, sonographic findings strongly suggested T21. In two no-call cases (Cases NC2 and NC3), an abnormal ultrasound with increased NT suggested T21 and T18, respectively. In Case NC1, an abnormal ultrasound suggested a monogenic disorder. In seven mosaic cases, sonographic findings with increased NT showed an abnormal karyotype, and two cases with normal NT eventually showed a normal karyotype. Finally, in all twenty-one discrepant cases, it is suggested that a fetal ultrasound, consistent with noninvasive or invasive genetic tests, is more reliable for proper prenatal management than NIPT or invasive tests without sonography.

## 5. Conclusions

This article describes the potential of rolling-circle replication as a powerful biosensing platform and the importance of examining the fetus in detail with ultrasound.

However, the potential applications foreseen by NIPT raise concerns that go beyond aneuploidy and its methodology. As its name suggests, NIPT is noninvasive, easy to perform, and accurate, but the importance of its results always involves ethical issues. Four key pillars have been proposed as an ethical framework for prenatal diagnosis: the purpose of screening; the proportionality; justice; and the social aspects [25]. It is also essential that specific issues related to cross-cultural backgrounds need to be considered [26]. In addition, from the ethical, legal, and social aspects, it is crucial to consider the right to autonomy, the stigmatization of, and discrimination against, people with disabilities, and the potential threats of sex-selective abortions [27]. Finally, we would like to add that NIPT should be conducted, taking into account the considerations mentioned above, from multiple perspectives.

## Figures and Tables

**Table 1:** Chorionic numbers and fetuses in 1218 cases.

Chorionic Numbers Detected by Sonography	Case Number	%
Singleton	1166	95.73%
DCDA twin	22	1.81%
MCDA twin	10	0.82%
MCDA vanishing twin with empty second amniotic sac	3	0.25%
DCDA vanishing twin with an empty sac	12	0.99%
DCDA vanishing twin with a nonviable fetus	5	0.41%
Total	1218	100%

DCDA; dichorionic diamniotic, MCDA; monochorionic diamniotic.

**Table 2:** Breakdown of the 184 abnormal genetic results.

Genetic Abnormality	Case Number	%
T21	60	32.6
T18	47	25.5
T13	12	6.5
T21/T18/T13 Mosaicism	7	3.8
Sex Chromosome aneuploidy	16	8.7
Triploidy	1	0.5
Aneuploidy Mosaicism*1	12	6.5
Structural abnormality*2	5	2.7
Structural Mosaicism*3	1	0.5
Pathogenic CNV*4	10	5.4
Single gene mutation*5	13	7.1
Total	184	100

T21; Trisomy 21, T18; Trisomy 18, T13; Trisomy 13, CNV; copy number variation. *1: 12 cases with 47,XXX/45,X, 47,XY,+2/45,X, 45,X/46,XX, 45,X/46,XY, 46,X,r(Y)(p11.3q12) /45,X,47,XX,+7/46,XX, 47,XX,+2/47,XX,+8/46,XX, 47,XY,+7/46,XY, 47,XX,+2/46,XX, 47,XY,+7/47,XY,+i(18)(p10) /48,XY,+i(18)(p10)×2/46,XY, 92,XXYY/46,XY, 47,XX,+9/46,XX. *2: two cases of 46,XY,del(3)(p12p14.1), one 45,XY,der(13;14)(q10;q10), one 46,XY,del(3)(p12p14.1), and one 46,XX,der(7)t(7;11)(q36;q14). *3: 46,XX,der(21)t(15;21)(q26.1;q22)/46,XX. *4: 10 cases with each CNV with 7p22.3q11.21(46,121–65,473,688)x3[0.23], 7q11.23q36.3(73,667,713–159,119,707)x3[0.25], Xp22.33(168,551–1,023,657)x1, 5q35.2q35.3(175,469,493–177,439,550)x1, 2q12.1q22.3(102,775,449–146,055,736) hmz, 2q32.1q34(184,130,386–213,573,573) hmz, 13q12.3q13.1(29,136,283–33,238,666)x1, 13q21.32q34(67,621,957–115,107,733)x3[0.22], 16q21q24.3(64,829,906–90,146,366) hmz, T16 mosaicism (10%) with mat UPD(16), 4p14p11(36,303,726–49,093,788)x2–3, 8q24.23(137,054,915–139,745,498)x1. *5: Four cases with PTPN11 mutation, two with COL1A2, one with each FGFR3, COL2A, L1CAM, N1PBL, RAF1, HRAS1, and SOX9 mutations.

**Table 3:** Sensitivity, specificity, positive predictive value, and negative predictive value for each trisomy in CRITO-NIPT study.

	Sensitivity	Specificity	PPV	NPV
T21	98.31% (58/59)	99.65% (1145/1149)	93.55% (58/62)	99.91% (1145/1146)
T18	100.00% (46/46)	99.48% (1156/1162)	88.46% (46/52)	100.00% (1156/1156)
T13	100.00% (12/12)	100.00% (1196/1196)	100.00% (12/12)	100.00% (1196/1196)

PPV; positive predictive value, NPV; negative predictive value.

**Table 4:** Genetic results and sonographic findings in CRITO false-positive (FP) and false-negative (FN) cases.

Case	CVS G–Band	CRITO Result	CRITO Z–Score	Placental Part						Cord	Membranes	Maternal Blood	Causal Factor of FP/FN Result	Increased NT	NT (mm)	Sonographic Findings
				A	B	C	D	E	F							
FP1	45,X	T21 positive	3.55	**MX+** **T21** **(16%)**	MX+D21	**MX+** **T21** **(8%)**	MX+D21	–	–	**MX+** **T21** **(4%)**		46,XX	CPM	+	8.8	Increased NT, CH, General edema, PE bilateral, Small NB, Tachycardia, Short FL/HL, Turner is strongly suspected
FP2	46,XX	T21 positive	4.18	D21	D21	D21	D21	D21	D21	D21	D21	46,XX	s/o nonviable twin with T21	–	2.2	TR mild, DCDA vanishing twin with a nonviable fetus
FP3	46,XY	T21 positive	7.52	D21	D21	D21	D21	D21	D21	D21	**T21** (14–74%) *	46,XX	nonviable twin with T21	–	1.4	Small NB, TR mild, DCDA vanishing twin with a nonviable fetus
FP4	46,XY	T21 positive	7.78	D21	D21	D21	D21	**T21** **(15%)**	D21	D21	D21	46,XX	CPM	–	2.2	Small NB, TR mild
FP5	46,XX	T18 positive	4.20	D18	D18	D18	D18	D18	D18	D18	D18	46,XX	unclear	–	2.1	TR mild, DV reverse
FP6	46,XX	T18 positive	12.76	D18	D18	D18	D18	D18	D18	D18	D18	46,XX	unclear	–	2.2	TR mild–moderate
FP7	46,XY	T18 positive	5.49	D18	D18	D18	D18	–	–	D18	D18	46,XX	unclear	–	1.6	TR mild
FP8	46,XY	T18 positive	3.56	D18	D18	D18	D18	D18	D18	D18	D18	46,XX	unclear	–	2.0	TR mild, SFD, SCH
FP9	46,XX	T18 positive	3.22	D18	D18	D18	D18	D18	D18	D18	D18	46,XX	unclear	–	1.2	no particular findings
FP10	46,XX	T18 positive	3.02	–	–	–	–	–	–	–	–	46,XX	unclear	–	1.8	TR mild
FN1	47,XY,+21	T21 negative	1.07	–	–	–	–	–	–	–	–	46,XX	unclear	+	7.2	Increased NT, GE mild, NB defect, Lowset ear, TR mild, Small stomach, DV reverse, Tachycardia, T21 is strongly suspected

* T21 was found only in a limited area of the membrane, Placentae could not be collected in Case FP10 and FN1, MX; monosomy X, D21: disomy of chromosome 21, D18: disomy of chromosome 18, CPM; confined placental mosaicism, NT; nuchal translucency, NB; nasal bone, FL; femur length, HL; humerus length, TR; tricuspid regurgitation, DV; ductus venosus, GE; general edema, DCDA; dichorionic diamniotic twin, IUFD; intrauterine fetal demise, SFD; small for date, SCH; subchorionic hemorrhage.

**Table 5:** Genetic results of placental sections and membranous sections in FP3.

Case FP3–Specimen	FISH		G–Band
	Disomy 21	Trisomy 21	
Placenta–A	100%	0%	46,XY
Placenta–B	100%	0%	culture failure
Placenta–C	100%	0%	46,XY
Placenta–D	100%	0%	46,XY
Placenta–E	100%	0%	46,XY
Placenta–E	100%	0%	46,XY
Cord	100%	0%	46,XY
Membranes–1	86%	**14%**	–
Membranes–2	90%	**10%**	–
Membranes–3	16%	**74%**	47,XY,+21
Membranes–4	100%	0%	–
Membranes–5	100%	0%	–

**Table 6:** CRITO validation results and sonographic findings in seven mosaicism (Mo) cases.

	Case	GA (Week + Day) for US and CVS	CVS			AC		CRITO–NIPT		Fetal Ultrasonography		
			QF–PCR	Uncultured FISH Aneuploidy (%)	G–Band Karyotype	Uncultured FISH Aneuploidy (%)	G–Band Karyotype	Result	Z–Score	Increased NT	NT Thickness (mm)	Sonographic Findings
Chromosome 21 relevant mosaicism	Mo1	13 + 3	XY,+21 Mosaicism	T21 (96%)	47,XY,+21 (100%)	–	–	T21 positive	22.05	+	4.6	Increased NT, GE very mild, Micrognathia, Lowset ear, DS like profile, Large VSD, TR moderate, Hyperechoic bowel, DV reverse, Tachycardia, Straight cord, T21 is strongly suspected
	Mo2	13 + 2	XX,+21 Mosaicism	Tetrasomy 21 (20%)	49,XX,+7,+21,+21 /46,XX (72%/28%)	–	–	T21 Negative	1.11	–	2.1	Small NB, Lowset ear, TR moderate
	Mo3	13 + 0	XXY	T21 (2%)	47,XXY /48,XXY,+21 (62%/38%)	T21 (2%)	47,XXY	T21 Negative	–0.48	+	3.4	Increased NT, Small NB, TR mild, SUA, Genetic disorder is suspected
	Mo4	13 + 3	XX,+21 Mosaicism	M21 (72%) T21 (8%)	45,XX,–21 (100%)	M21 (1%) T21 (91%)	46,XX,i(21)(q10)	T21 positive	10.87	+	5.2	Increased NT, Small NB, Micrognathia, Lowset ear, TR mild, DV defect, T21 is strongly suspected
Chromosome 18 relevant mosaicism	Mo5	13 + 5	XX,+18 Mosaicism	T18 (56%)	47,XX,+18/46,XX (44%/56%)	–	–	T18 Negative	2.09	+	8.3	Increased NT, GE moderate, Small NB, Micrognathia, Lowset ear, T18 like profile, Wrist contracture bilateral, Cardiomegaly, Large VSD, TR severe, MR severe, Stomach invisible, Hyperechoic bowel, DV reversed flow, SUA, Umb.A.reverse, T18 is strongly suspected,
	Mo6	13 + 0	XY,T18 Mosaicism	T18 (88%)	47,XY,+18 (100%)	–	–	T18 positive	7.05	+	2.9	Increased NT, NB defect, T18 like profile, Micrognathia, Lowset ear, Hypoplastic ear, Wrist contracture bilateral, Contracted lower extremities, Club foot (right), s/o DORV, RV>LV, TR moderate–severe, Omphalocele containing only bowel, DV reversed flow, FGR, T18 is strongly suspected
Chromosome 13 relevant mosaicism	Mo7	12 + 3	Normal XY	T13 (6%)	46,XY (100%)	T13 (0%)	T13 (0%)	T13 Negative	1.25	–	2.0	TR mild

GA; gestational age, CVS; chorionic villus sampling, AC; amniocentesis, NT; nuchal translucency, NB; nasal bone, GE; general edema, DS; Down syndrome, VSD, ventricular septal defect, TR; tricuspid regurgitation, DV; ductus venosus, SUA; single umbilical artery, MR; mitral regurgitation, Umb.A; umbilical artery, DORV; double outlet right ventricle, RV; right ventricle, LV; left ventricle, FGR; fetal growth restriction, T21; trisomy 21, M21; monosomy 21, T18; trisomy 18, T13; trisomy 13.

**Table 7:** Genetic test results for each sample in Case Mo2.

Case Mo2–Specimen	QF–PCR	FISH			G–Band
		Trisomy 7 + Tetrasomy 21	Disomy 7 + Disomy 21	Disomy 7 + Monosomy 21	
CVS	s/o T21 mosaicism	20%	80%	0%	49,XX,+7,+21,+21/46,XX (72%/28%)
Placenta–A	s/o T21 mosaicism	24%	76%	0%	46,XX (100%)
Placenta–B	s/o T21 mosaicism	0%	90%	10%	49,XX,+7,+21,+21/46,XX (15%/85%)
Placenta–C	s/o T21 mosaicism	10%	80%	10%	49,XX,+7,+21,+21/45,XX,–21/46,XX (5%/10%/85 %)
Placenta–D	s/o T21 mosaicism	20%	70%	10%	49,XX,+7,+21,+21/46,XX (10%/90%)
Placenta–E	s/o T21 mosaicism	24%	64%	12%	49,XX,+7,+21,+21/46,XX (10%/90%)
Cord	Normal XX	0%	100%	0%	46,XX (100%)
Membranes	Inconclusive	20%	74%	6%	49,XX,+7,+21,+21/46,XX (5%/95%)
Cord blood	Normal XX	0%	100%	0%	46,XX (100%)

**Table 8:** Genetic test results for each sample in Case Mo4.

Case Mo4–Specimen	QF–PCR	FISH			G–Band
		Monosomy 21	Disomy 21	Trisomy 21	
CVS	s/o low level T21 mosaicism	72%	20%	8%	45,XX,–21 (100%)
Amniotic cell	T21	1%	8%	91%	46,XX,i(21)(q10) (100%)
Placenta–A	s/o low level T21 mosaicism	56%	10%	34%	45,XX,–21/46,XX,i(21)(q10) (95.5%/4.5%)
Placenta–B	s/o middle level T21 mosaicism	68%	18%	14%	
Placenta–C	s/o low level T21 mosaicism	60%	14%	26%	
Placenta–D	s/o low level T21 mosaicism	64%	8%	28%	
Cord	s/o high level T21 mosaicism	6%	8%	86%	46,XX,i(21)(q10) (100%)
Membranes	s/o high level T21 mosaicism	34%	16%	50%	45,XX,–21/46,XX,i(21)(q10) (2%/98%)
Maternal blood	normal	–	–	–	46,XX
Paternal blood	normal	–	–	–	46,XY

CVS; chorionic villus sampling, QF-PCR; quantitative fluorescence polymerase chain reaction, FISH; fluorescence in situ hybridization, 45,XX,–21; Monosomy 21, 46,XX,i(21)(q10); Robertsonian Trisomy 21 consisted of maternal isochromosome 21 and paternal 21 chromosome, s/o; suspect of.

**Table 9:** CRITO results and sonographic findings in three no call (NC) cases.

Case	GA	Invasive Procedure	BMI	G–Band Result	CRITO-NIPT	Increased NT	NT (mm)	Sonographic Findings
NC1	30w0d	AC	21.1	46,XY,del(13)(q13q21.3)	No Call	–	–	Thick prenasal skin, Low nasal bridge, Small NB, Micrognathia, Hypertrichosis, TR, Undescendant testis (left), Small mid phalanx of 5th digit (bilateral), Large head, Increased AF (AFI 23.68cm), T21 or Genetic disorder such as Costello or Cornelia de lange syndrome is suspected
NC2	12w2d	CVS	22.8	47,XY,+21	No Call	+	5.3	Increased NT, Small CH, GE, bilateral, PE, NB defect, Micrognathia, Lowset ear, s/o Large VSD, TR moderate, Levocardia, RV>LV, Hyperechoic bowel, DV reverse, Short FL, T 21 is strongly suspected
NC3	13w1d	CVS	25.8	47,XY,+18	No Call	+	10.6	Increased NT, CH, GE, Small NB, Micrognathia, Lowset ear, Hypoplastic ear, Cleft lip (left), Maxillary gap, Mild wrist contracture bilateral, RV>LV, TR, DV reverse, Bradycardia, T18 is strongly suspected

GA; gestational age, AC; amniocentesis, CVS; chorionic villus sampling, BMI; body mass index, NT; nuchal translucency, NB; nasal bone, CH; cystic hygroma, GE; general edema, TR; tricuspid regurgitation, DV; ductus venosus, AF; amniotic fluid, AFI; amniotic fluid index, PE; pleural effusion, VSD; ventricular septal defect, RV; right ventricle, LV; left ventricle, T21; trisomy 21, T18; trisomy 18.

## Data Availability

The datasets presented in this study are available from the corresponding author. The data are not publicly available out of respect for individual privacy.
